# A high proliferation rate measured by cyclin A predicts a favourable chemotherapy response in soft tissue sarcoma patients

**DOI:** 10.1038/sj.bjc.6690801

**Published:** 1999-11

**Authors:** R L Huuhtanen, T A Wiklund, C P Blomqvist, T O Böhling, M J Virolainen, B Tribukait, L C Andersson

**Affiliations:** Department of Oncology, Helsinki University Central Hospital, PO Box 180, Helsinki, FIN-00029 HUCS; Department of Pathology, University of Helsinki, Haartman Institute and Helsinki University Central Hospital, Helsinki, Finland; Department of Radiobiology and Pathology, Karolinska Institute and Hospital, Stockholm, Sweden

**Keywords:** soft tissue sarcoma, proliferation, chemotherapy response, cyclin A, Ki-67, S phase fraction

## Abstract

A small but not insignificant number of patients experience a prolonged survival after treatment of metastatic soft tissue sarcoma. This must be weighed against the majority of the patients who benefit little from the therapy, but nevertheless experience its side-effects. It would therefore be of utmost importance to be able to screen for those patients who respond to the treatment. Since proliferating cells are more sensitive to chemotherapy than non-proliferative cells, we measured the proliferation rate of the primary tumour of 55 soft tissue sarcoma patients with locally advanced or metastatic disease by determining the flow cytometric S phase fraction and immunohistochemical Ki-67 and cyclin A scores. S phase fraction or Ki-67 score did not predict chemotherapy response or progression-free survival. A high cyclin A score, however, correlated with a better chemotherapy response (*P* = 0.02) and longer progression-free survival time (*P* = 0.04). Our results suggest that a high cyclin A score predicts chemotherapy sensitivity. © 1999 Cancer Research Campaign


*British Journal of Cancer* (1999) **81**(6), 1017–1021
© 1999 Cancer Research Campaign
Article no. bjoc.1999.0801

### A high proliferation rate measured by cyclin A predicts a favourable chemotherapy response in soft tissue sarcoma patients

**RL Huuhtanen[1], TA Wiklund[1], CP Blomqvist[1], TO Böhling[2], MJ Virolainen[2], B Tribukait[3] and LC Andersson[2,3]**

[1]Helsinki University Central Hospital, Department of Oncology, PO Box 180, Helsinki, FIN-00029 HUCS, [2]University of Helsinki, Haartman Institute and Helsinki University Central Hospital, Department of Pathology, Helsinki, Finland; [3]Karolinska Institute and Hospital, Department of Radiobiology and Pathology; Stockholm, Sweden

**Summary** A small but not insignificant number of patients experience a prolonged survival after treatment of metastatic soft tissue sarcoma. This must be weighed against the majority of the patients who benefit little from the therapy, but nevertheless experience its side-effects. It would therefore be of utmost importance to be able to screen for those patients who respond to the treatment. Since proliferating cells are more sensitive to chemotherapy than non-proliferative cells, we measured the proliferation rate of the primary tumour of 55 soft tissue sarcoma patients with locally advanced or metastatic disease by determining the flow cytometric S phase fraction and immunohistochemical Ki-67 and cyclin A scores. S phase fraction or Ki-67 score did not predict chemotherapy response or progression-free survival. A high cyclin A score, however, correlated with a better chemotherapy response ($P = 0.02$) and longer progression-free survival time ($P = 0.04$). Our results suggest that a high cyclin A score predicts chemotherapy sensitivity. © 1999 Cancer Research Campaign

**Keywords:** soft tissue sarcoma; proliferation; chemotherapy response; cyclin A; Ki-67; S phase fraction

The response rate to combination chemotherapy (CT) of advanced soft tissue sarcoma (STS) is 27–45%, with complete response (CR) rate around 10% (Schutte et al, 1990; Steward et al, 1993; Santoro et al, 1995; Saeter et al, 1997). Of all patients treated with CT, 15% were longtime survivors and 5–6% could possibly be cured (Benjamin, 1998; Wiklund et al, 1997).

In order to gain prolonged survival in a few individuals, many must be exposed to the side-effects of CT. A test able to identify those patients who are going to benefit from CT would therefore be of great benefit. It is known that the performance status affects CT response (Borden et al, 1990). However, little is known about tumour-related factors which might predict CT response in STS. In several other tumours a high proliferation rate has been shown to be one of the factors increasing responsiveness to CT (Mattern et al, 1986; Remvikos et al, 1989; O'Reilly et al, 1992; Hietanen et al, 1995).

Several methods are available to measure the proliferation activity of tumour cells. Determination of the tumour nuclear antigen Ki-67 and S phase fraction (SPF) by flow cytometry are two of the most studied ones (Gerdes et al, 1984; Hedley, 1989). In recent years knowledge concerning the regulation of the cell cycle has increased. Cyclins together with cyclin dependent kinases (cdk) control the cell cycle by phosphorylation. Every cyclin has its specific time of appearance in the cell cycle. Cyclin A is essential for the DNA replication in the S phase and it is active during the initiation of mitosis. There are very few reports on cyclin A as a marker of proliferative cell fraction in cancer. In two recent studies by Volm, tumour tissue negativity for cyclin A expression predicted a favourable outcome in non-small-cell lung cancer patients (Volm et al, 1997, 1998). We are aware of no previous studies correlating cyclin A score of the tumour to chemotherapy response.

Studies on breast cancer have shown that patients whose primary tumour has high proliferative activity respond better to CT (Remvikos et al, 1989; O'Reilly et al, 1992; Hietanen et al, 1995). Similar results have been obtained in a melanoma study (Karlsson et al, 1996). Only one study, as far as we know, has investigated the prediction of CT response in STS patients (Schmidt et al, 1993). In this study there was a tendency towards better histopathologically verified CT response if the tumour had a high SPF, although the result was not statistically significant.

The purpose of the present study was to find out whether tumour cell proliferation could predict CT response and outcome in STS patients. We collected the clinical data and evaluated the CT responses in 75 STS patients treated in our institution by the same combination CT regimen (IADIC). The proliferation rate of the primary tumour samples was determined by flow cytometric SPF and immunohistochemically by Ki-67 and cyclin A scores.

### PATIENTS

Consecutive patients treated between June 1988 and January 1997 at the Department of Oncology, Helsinki University Central Hospital, with the IVADIC regimen were included in the study. Twenty-eight of these patients were included in a previously published phase II CT study (Wiklund et al, 1992). The CT schedule is shown in Table 1. Since October 1995 vincristine was deleted from the schedule (IADIC).

Twenty cases were excluded from the study, eight due to unavailable histological samples; the response rate in these cases

*Received 30 November 1998*
*Revised 21 April 1999*
*Accepted 30 April 1999*

*Correspondence to:* RL Huuhtanen

**Table 1** IADIC treatment schedule

| Agent | Dose | Schedule |
|---|---|---|
| Ifosfamide | 1 $g/m^2$/day i.v. | days 1–5 |
| Vincristine[a] | 1.5 $mg/m^2$ i.v. | day 1 |
| Doxorubicin | 50 $mg/m^2$ i.v. | day 1 |
| Dacarbazine | 250 $mg/m^2$/day i.v. | days 1–5 |
| Mesna | 0.2 $g/m^2 \times 3$/day i.v. | days 1–5 |

i.v. = intravenously. [a]Vincristine was deleted from the schedule in October 1995.

was 50% (4/8). Twelve cases were excluded since the CT response was not evaluable. One patient had received radiotherapy to all measurable lesions just prior to CT, one was given early radiotherapy during the treatment, the lesions were not measurable in two cases, two cases had poor performance status and did not receive the scheduled treatment, in two cases the documentation of the response was insufficient, and in four cases the patient received only one CT cycle due to the patients' refusal or complications.

The pretreatment characteristics of the 55 patients included in the study are shown in Table 2. In 42 cases all three proliferation measurements were available (SPF $n$ = 50, Ki-67 score $n$ = 47 and cyclin A score $n$ = 48).

The median time from diagnosis to beginning of CT was 335 days (range 6–4609 days). The number of patients still alive was 19, and seven of them were disease-free. The median follow-up time of the living patients was 18 months (range 7–85 months). Thirty-six patients died of their disease.

### METHODS

The CT response evaluation was made retrospectively according to Miller et al (1981). RH made the response evaluation by reviewing patients' documents and diagnostic images. CR and partial responses (PR) were also evaluated by TW. Only patients with stable disease for at least 3 months were classified into the no change (NC) group. Some patients were given radiotherapy, underwent surgery or changed the CT schedule before objective signs of progression were recorded. In these patients the follow-up for progressive disease (PD) was cut off at the first day of surgery or radiotherapy, or beginning of a new CT schedule.

Formalin-fixed, paraffin-embedded primary tumour samples were collected. Sample deparaffination was done in xylene and rehydration in alcohol to distilled water. Antigen demasking was carried out by heating the samples in a microwave oven (850 W) in citric acid buffer (pH 6, 0.1 M) for 20 min, buffer was added in need. Treatment with 1.6% methanolperoxidase was used to inhibit endogenous peroxidase activity. For immunohistochemistry, the specimens were incubated overnight at room temperature with 1:100 diluted mouse monoclonal antibody to human cyclin A (Novocastra), and for Ki-67 determination with 1:500 mouse anti-human monoclonal Mib-1 antibody (PharMingen). The binding of the primary antibody was detected by a peroxidase-conjugated secondary antibody using the Vectastain® ABC kit (Vector Laboratories Inc.). The sections were counterstained with haematoxylin. As positive and negative controls for cyclin A stainings, we used hyperplastic tonsil tissue in cyclin A stainings. The primary antibody was omitted from the negative controls.

**Table 2** Pretreatment characteristics of the patients and chemotherapy response rates according to the pretreatment characteristics

| | *n* (%) | Response rate % |
|---|---|---|
| Sex | | |
| Female | 33 (60) | 52 |
| Male | 22 (40) | 36 |
| WHO performance status | | |
| 0 | 27 (49) | 52 |
| 1 | 26 (47) | 38 |
| 2 | 2 (4) | 0 |
| Age | | |
| Median (range), years | 50 (16–70) | |
| Primary location | | |
| Visceral | 21 (38) | 48 |
| Limb | 22 (40) | 55 |
| Trunk | 6 (11) | 17 |
| Head and neck | 3 (5.5) | 67 |
| Unknown | 3 (5.5) | 0 |
| Stage of disease | | |
| Metastatic disease | 36 (66) | 44 |
| Primary tumour and metastasis | 5 (9) | 60 |
| Local recurrence | 4 (7) | 75 |
| Local recurrence and metastasis | 10 (18) | 30 |
| Metastasis site | | |
| Pulmonary | 29 (57) | 48 |
| Pulmonary and other | 15 (29) | 33 |
| Other without pulmonary | 7 (14) | 71 |
| Ploidy | | |
| Diploid | 30 (59) | 40 |
| Non-diploid | 21 (41) | 43 |
| Grade | | |
| Low (1–2) | 9 | |
| High (3–4) | 46 | |
| Histology | | |
| Malignant fibrous histiocytoma | 11 | 56 |
| Liposarcoma | 4 | 50 |
| Leiomyosarcoma[a] | 13 | 62 |
| Gastrointestinal stromal cell tumour | 3 | 33 |
| Synovial sarcoma | 6 | 33 |
| Extraskeletal Ewing's sarcoma | 2 | 100 |
| Fibrosarcoma | 2 | 0 |
| Unclassified sarcoma | 7 | 14 |
| Other sarcomas[b] | 4 | 25 |
| Rhabdomyosarcoma | 3 | 67 |

[a]Six uterine primaries. [b]Cystosarcoma phylloides malignum, epithelioid sarcoma, angiosarcoma, malignant schwannoma.

We chose the tumour area with the highest density of positive nuclear staining for quantification of the immunostaining. To calculate the percentage of positively stained nuclei, an ocular grid of 100 (10 × 10) squares was used at 10 × 40 magnification. All positive nuclei from this area were counted. To estimate the negative nuclei in the same area of 100 squares, three different rows of 10 squares were counted and the mean score multiplied by 10. In the case of tumours of scarce cellularity, several fields were evaluated, and negative nuclei were counted from the whole grid area of a hundred squares. The percentage of positive nuclei was counted by dividing the number of the positive stained cells by the entire number of cells in the same area. All samples were scored by two independent observers (RH and TB). In the case of more than 5% interobserver difference in the result, the sample was rescored by the two investigators together.

 © 1999 Cancer Research Campaign

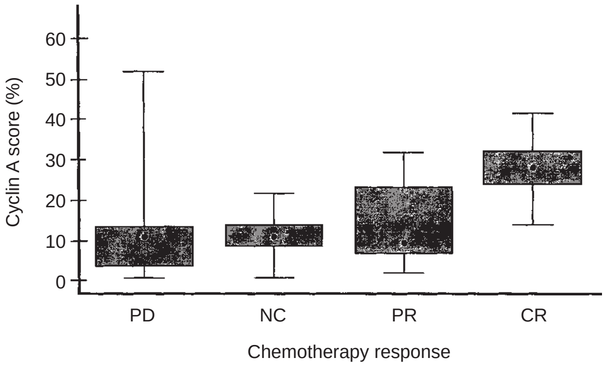

**Figure 1** The median cyclin A scores, ranges and quartiles in different chemotherapy response groups

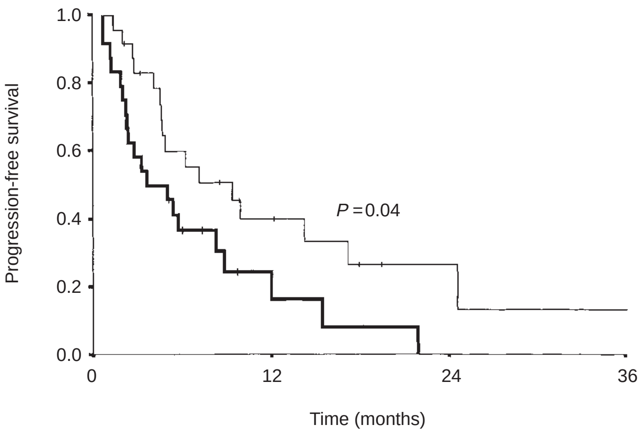

**Figure 2** Progression-free survival time. The thin line indicates patients whose cyclin A score is above the median (11.5%) and the bold line shows the cyclin A scores below or equal to the median

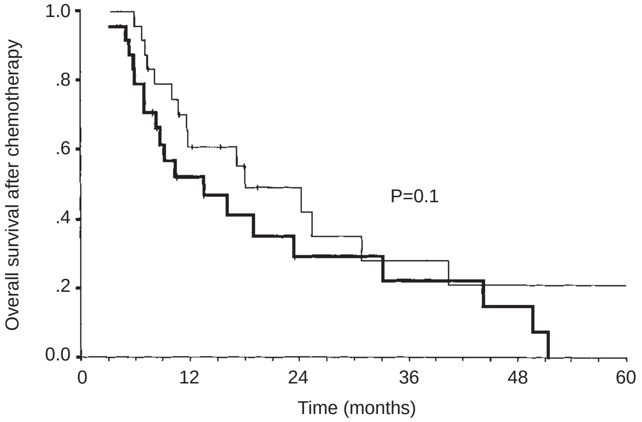

**Figure 3** Overall survival time. The thin line indicates patients whose cyclin A score is above the median (11.5%) and the bold line shows the cyclin A scores below or equal to the median

In flow cytometric measurements, a modification of the basic Hedley method was used (Hedley, 1989; Heiden et al, 1991). The sections (100 μm) from the paraffin blocks were placed into fine-mesh bags which were inserted into cassettes and then dewaxed and rehydrated in a tissue processor. Enzymatic digestion was done with Subtilisin Carlsberg (Sigma Protease type 24) and all centrifugation steps were omitted, resulting in nuclei suspensions with extremely low amounts of clumped nuclei and debris. The nuclei were stained with DAPI (final concentration 5 μmol) and analysed using a PAS 2 flow cytometer. To confirm the ploidy of near diploid populations, the DNA content of identified nuclei was measured by means of a static image analysis system. Tumours were considered diploid if only one G1-peak was present. For determination of the DNA index in grossly aneuploid cell populations, the first peak to the left was assumed to represent diploid cells. For the determination of the percentage of cells in the S phase of the cell cycle, the MultiCycle program with the sliced-nuclei option for background subtraction developed by Rabinowich was used (Phoenix Flow System, San Diego, CA, USA).

### Statistical methods

The Macintosh Statistica® program was used for most statistical analyses. Correlations between ordinal variables were calculated with Spearman rank order correlation test. Progression-free survival (PFS) and overall survival (OS) analysis were calculated with the Cox regression model. Proliferative indexes were used as continuous variables. PFS and OS curves were calculated by the Kaplan–Meier model by the Macintosh SPSS® program. PFS, OS after CT and disease-free time after CR have been calculated from the beginning of CT.

### RESULTS

Eight patients (14%) had a CR, 17 (31%) PR, 18 (33%) had NC and 12 (22%) PD. The response rates according to pretreatment characteristics are shown in Table 1. The median disease-free time in completely responding patients was 11 months (range 9–85 months).

The median cyclin A was 11.5% (range 1–52%). The median S phase was 5.25% (range 0.4–30.9%). The median SPF of diploid tumours was 4.5% (range 0.4–22.5%) and in non-diploid tumours 11.7% (range 2.8–30.9%). Median Ki-67 was 19% (range 2–45%). Table 3 shows the number of patients in each response class divided according to the median of cyclin A score, SPF and Ki-67 score. The medians and ranges of cyclin A score, SPF and Ki-67 score in each response class are shown in Table 4. Figure 1 shows the median cyclin A scores, ranges and quartiles according to CT response.

A significantly higher CT response rate was found in tumours with high cyclin A score ($P = 0.02$), while no significant correlation was found for SPF or Ki-67 score ($P = 0.3$ and $P = 0.2$ respectively). SPF or Ki-67 score did not predict PFS ($P = 0.75$ and $P = 0.2$ respectively) or OS ($P = 0.76$ and $P = 0.7$ respectively). The median PFS was 3.7 months when the primary tumour cyclin A score was below the median, vs. 9.3 months when the cyclin A score was above the median ($P = 0.04$) (Figure 2). The median OS time from the beginning of CT was 13.4 months when the cyclin A score was below the median, and 18 months when the cyclin A score was above the median ($P = 0.1$) (Figure 3).

### DISCUSSION

We have demonstrated that an immunohistochemically determined high cyclin A score predicted a better CT response and a longer

*© 1999 Cancer Research Campaign* 

**Table 3** Number of patients and percentages in the groups below and above the median of cyclin A, SPF and Ki-67

| | PD (%) | NC (%) | PR (%) | CR (%) | Responder/all |
|---|---|---|---|---|---|
| Cyclin A | 11 | 15 | 16 | 6 | 22/48 |
| Cyclin A ≤ 11.5% | 6 (25) | 9 (37.5) | 9 (37.5) | 0 (0) | 9/24 |
| Cyclin A > 11.5% | 5 (21) | 6 (25) | 7 (29) | 6 (25) | 13/24 |
| SPF | 11 | 18 | 14 | 7 | 21/50 |
| SPF ≤ 5.2% | 6 (24) | 10 (40) | 7 (28) | 2 (8) | 9/25 |
| SPF > 5.2% | 5 (20) | 8 (32) | 7 (28) | 5 (20) | 12/25 |
| Ki-67 | 9 | 15 | 15 | 8 | 23/47 |
| Ki-67 ≤ 19% | 6 (25) | 8 (33.5) | 8 (33.5) | 2 (8) | 10/24 |
| Ki-67 > 19% | 3 (13) | 7(30) | 7 (30) | 6 (27) | 13/23 |

**Table 4** Medians and ranges of cyclin A, Ki-67 and SPF in different chemotherapy response groups

| | PD | NC | PR | CR |
|---|---|---|---|---|
| Cyclin A median | 11 | 11 | 9.5 | 28.3 |
| (range) | (1–52) | (1–22) | (2–32) | (14–41.5) |
| Ki-67 median | 15 | 19 | 18 | 26 |
| (range) | (6.5–33.5) | (2–43) | (3–45) | (6–38) |
| SPF median | 4.7 | 5.5 | 4.6 | 15.8 |
| (range) | (0.4–30.9) | (1.7–29.3) | (1.3–19.7) | (3–22.5) |

PFS in patients with advanced STS. All the patients who reached CR had a cyclin A score above the median.

Although the Ki-67 score and SPF did not predict CT response or progression-free survival in this material, there was a tendency for those patients who reached CR to have more often a Ki-67 score or SPF above the median than those who did not. Due to our limited sample size, which diminished the statistical power, the moderate predictive power of these two proliferation measurements cannot be excluded. Because STS is a rare disease, the possibility of collecting a larger sample size is limited.

The longer time to progression after CT is noteworthy since in our previous study patients whose primary tumour had a high cyclin A score had shorter OS and time to metastasis after primary treatment than those patients whose primary tumour had a low cyclin A score, and only six out of 126 (5%) patients received adjuvant chemotherapy (Huuhtanen et al, 1999). This suggests a prominent effect on the natural course of the disease by CT in patients whose tumours have a fast proliferation rate. A similar correlation between a high proliferation rate and improvement of prognosis by CT has previously been noted in other malignancies including breast cancer, melanoma and lymphoma. (Joensuu et al, 1994; Stål et al, 1994; Hahka-Kemppinen et al, 1997).

The CR group differs clearly from the other patients by the significantly higher median values of cyclin A score, SPF and Ki-67 score. The PR group did not show such difference. The study by Wiklund et al (1997) suggested that the group of patients with clinical PR is heterogenous. When surgical resection of metastasis was performed after CT, clinical PR correlated poorly with the histopathological CT response (Wiklund et al, 1997). In the smaller study by Schmidt et al (1993), the correlation with clinical and histopathological CT response in STS was similar in 70% of the cases.

We have previously shown that cyclin A, Ki-67 score and SPF correlate strongly to each other. However, cyclin A gave more information about the prognosis compared to SPF and Ki-67 score (Huuhtanen et al, 1999). The present investigation indicates that cyclin A score predicted the clinical behaviour of the disease better than the two other proliferation markers, also with respect to the response to cytotoxic treatment. These different markers of proliferation reflect the different phases of the cell cycle. As cyclin A is active from the beginning of S phase to the beginning of mitosis, it reveals the cell cycle phases most sensitive to CT drugs (Mattern et al, 1986; McLaughlin, 1994). In the study by Volm, breast cancer cell lines with high cyclin A were significantly more sensitive to doxorubicin than cell lines with low cyclin A activity (Volm et al, 1997). As far as we know, the present study is the first one to demonstrate a significant tumour-related predictive factor for STS, as well as being the first study of cyclin A and CT response in any malignancy.

It has been debated whether patients with metastatic STS should be treated at all by CT (Rouesse and Bourgeois, 1998). While a CR of metastatic STS to CT in many cases is associated with a long survival, perhaps even complete recovery in some patients, the proportion of CR with the present drug combinations is disappointingly low (Wiklund et al, 1997; Benjamin, 1998). Thus any method able to select patients with tumours which potentially are sensitive to CT would be of great clinical value. Based on the present study, proliferation determination by cyclin A score is a promising tool for gaining more information in the selection of different STS treatment options. Since a high cyclin A score is predictive for a favourable chemotherapy response as well as for an increased risk of relapse in primary disease it might also be useful for choosing patients with STS benefiting from adjuvant chemotherapy; a more extensive material would clarify the subject.

### ACKNOWLEDGEMENTS

This study was supported by grants from the Finnish Cancer Foundation, Finska Läkaresällskapet (Finnish Medical Society), Albin Johansson Foundation, and by a state subsidy for research and development at Helsinki University Central Hospital. We thank Ms Pirjo Jutila for technical assistance.

### REFERENCES

Benjamin RS (1998) Current controversies in cancer: should patients with advanced sarcomas be treated with chemotherapy: Pro. *Eur J Cancer* **34**: 958–962

Borden EC, Amato DA, Edmonson JH, Ritch PS and Shiraki M (1990) Randomized comparison of doxorubicin and vindesine to doxorubicin for patients with metastatic soft-tissue sarcomas. *Cancer* **66**: 862–867

Gerdes J, Lemke H, Baisch H, Wacker HH, Schwab U and Stein H (1984) Cell cycle analysis of a cell proliferation-associated human nuclear antigen defined by the monoclonal antibody Ki-67. *J Immunol* **133**: 1710–1715

Hahka-Kemppinen M, Muhonen T, Nordling S and Pyrhönen S (1997) DNA flow cytometry and the outcome of chemoimmunotherapy in metastatic melanoma. *Melanoma Res* **7**: 329–334

Hedley DW (1989) Flow cytometry using paraffin-embedded tissue: five years on. *Cytometry* **10**: 229–241

Heiden T, Wang N and Tribukait B (1991) An improved Hedley method for preparation of paraffin-embedded tissues for flow cytometric analysis of ploidy and S-phase. *Cytometry* **12**: 614–621

Hietanen P, Blomqvist C, Wasenius VM, Niskanen E, Franssila K and Nordling S (1995) Do DNA ploidy and S-phase fraction in primary tumour predict the response to chemotherapy in metastatic breast cancer? *Br J Cancer* **71**: 1029–1032

Huuhtanen RL, Blomqvist CP, Böhling TO, Wiklund TA, Tukiainen EJ, Virolainen M, Tribukait B and Andersson LC (1999) Expression of cyclin A in soft tissue sarcomas correlates with tumor aggressiveness. *Cancer Res* **59**: 2885–2890

 *© 1999 Cancer Research Campaign*

Joensuu H, Ristamäki R, Söderström KO and Jalkanen S (1994) Effect of treatment on the prognostic value of S-phase fraction in non-Hodgkin's lymphoma. *J Clin Oncol* **12**: 2167–2175

Karlsson M, Jungnelius U, Aamdal S, Boeryd B, Carstensen J, Kågedal B, Westberg R and Wingren S (1996) Correlation of DNA ploidy and S-phase fraction with chemotherapeutic response and survival in a randomized study of disseminated malignant melanoma. *Int J Cancer* **65**: 1–5

Mattern J, Wayss K and Volm M (1986) Predicting chemosensitivity of tumors. *Breast Cancer Res Treat* **8**: 157–159

McLaughlin CL (1994) Principles of chemotherapy. In: *Practical Oncology,* Cameron RB (ed.), pp. 9–11. Prentice-Hall: Conneticut

Miller AB, Hoogstraten B, Staquet M and Winkler A (1981) Reporting results of cancer treatment. *Cancer* **47**: 207–214

O'Reilly SM, Camplejohn RS, Rubens RD and Richards MA (1992) DNA flow cytometry and response to preoperative chemotherapy for primary breast cancer. *Eur J Cancer* **28**: 681–683

Remvikos Y, Beuzeboc P, Zajdela A, Voillemot N, Magdelenat H and Pouillart P (1989) Correlation of pretreatment proliferative activity of breast cancer with the response to cytotoxic chemotherapy. *J Natl Cancer Inst* **81**: 1383–1387

Rouesse J and Bourgeois H (1998) Current controversies in cancer: should patients with advanced sarcomas be treated with chemotherapy: contra. *Eur J Cancer* **34**: 962–964

Saeter G, Alvegård TA, Monge OR, Strander H, Turesson I, Klepp R, Söderberg M, Wist E, Raabe N, Erlanson M, Solheim OP and Hannisdal E (1997) Ifosfamide and continuous infusion etoposide in advanced adult soft tissue sarcoma. A Scandinavian Sarcoma Group Phase II Study. *Eur J Cancer* **33**: 1551–1558

Santoro A, Tursz T, Mouridsen H, Verweij J, Steward W, Somers R, Buesa J, Casali P, Spooner D, Rankin E and et al (1995) Doxorubicin versus CYVADIC versus doxorubicin plus ifosfamide in first-line treatment of advanced soft tissue sarcomas: a randomized study of the European Organization for Research and Treatment of Cancer Soft Tissue and Bone Sarcoma Group. *J Clin Oncol* **13**: 1537–1545

Schmidt RA, Conrad E3, Collins C, Rabinovitch P and Finney A (1993) Measurement and prediction of the short-term response of soft tissue sarcomas to chemotherapy. *Cancer* **72**: 2593–2601

Schutte J, Mouridsen HT, Stewart W, Santoro A, van Oosterom A, Somers R, Blackledge G, Verweij J, Dombernowsky P, Thomas D and et al (1990) Ifosfamide plus doxorubicin in previously untreated patients with advanced soft tissue sarcoma. The EORTC Soft Tissue and Bone Sarcoma Group. *Eur J Cancer* **26**: 558–561

Stål O, Skoog L, Rutqvist LE, Carstensen JM, Wingren S, Sullivan S, Andersson AC, Dufmats M and Nordenskjöld B (1994) S-phase fraction and survival benefit from adjuvant chemotherapy or radiotherapy of breast cancer. *Br J Cancer* **70**: 1258–1262

Steward WP, Verweij J, Somers R, Spooner D, Kerbrat P, Clavel M, Crowther D, Rouesse J, Tursz T, Tueni E and et al (1993) Granulocyte-macrophage colony-stimulating factor allows safe escalation of dose-intensity of chemotherapy in metastatic adult soft tissue sarcomas: a study of the European Organization for Research and Treatment of Cancer Soft Tissue and Bone Sarcoma Group. *J Clin Oncol* **11**: 15–21

Volm M, Koomagi R, Mattern J and Stammler G (1997) Cyclin A is associated with an unfavourable outcome in patients with non-small-cell lung carcinomas. *Br J Cancer* **75**: 1774–1778

Volm M, Rittgen W and Drings P (1998) Prognostic value of ERBB-1, VEGF, cyclin A, FOS, JUN and MYC in patients with squamous cell lung carcinomas. *Br J Cancer* **77**: 663–669

Wiklund T, Saeter G, Strander H, Alvegård T and Blomqvist C (1997) The outcome of advanced soft tissue sarcoma patients with complete tumour regression after either chemotherapy alone or chemotherapy plus surgery. The Scandinavian Sarcoma Group experience. *Eur J Cancer* **33**: 357–361

Wiklund TA, Blomqvist CP, Virolainen M and Elomaa I (1992) Ifosfamide, vincristine, doxorubicin and dacarbazine in adult patients with advanced soft-tissue sarcoma. *Cancer Chemother Pharmacol* **30**: 100–104

*© 1999 Cancer Research Campaign*
